# MAPSeg: self-supervised colorectal polyp segmentation via memory-augmented framework and synthetic polyp simulation

**DOI:** 10.3389/fdgth.2026.1797571

**Published:** 2026-05-14

**Authors:** Claudia Delprete, Domenico Buongiorno, Roberto Maria Scardigno, Elena Sibilano, Antonio Brunetti, Vitoantonio Bevilacqua

**Affiliations:** Department of Electrical and Information Engineering, Polytechnic University of Bari, Bari, Italy

**Keywords:** anomaly detection, medical image analysis, memory-augmented networks, polyp segmentation, synthetic data generation, unsupervised learning

## Abstract

**Introduction:**

Colorectal cancer originates in most cases from polyps that progressively become malignant over time and colonoscopy offers a highly studied early diagnostic strategy to ensure a timely treatment plan. Automatic image segmentation of polyps, using intelligent supervised approaches, achieved good performance, but the need of their large annotated datasets limits the clinical applicability.

**Methods:**

This study presents MAPSeg (Memory-Augmented Polyp Segmentation), a fully self-supervised and annotation-free framework for colorectal polyp segmentation, trained exclusively on images of healthy mucosa within an anomaly detection paradigm. The key novelty of MAPSeg mainly lies in SIMPO (Simulation of Polyps), a synthetic augmentation strategy that generates realistic polyp shapes and textures in a colon-specific context, combined with a memory-augmented encoder that models structural priors of normal tissue.

**Results:**

Extensive experiments demonstrate that MAPSeg outperforms the strongest unsupervised methods by approximately 23% in Intersection over Union and 12% in DICE score on the Hyper-Kvasir dataset and consistently maintains this performance margin across multiple out-of-distribution benchmarks, indicating strong generalization capability.

**Discussion:**

The results highlight MAPSeg supported by SIMPO as a viable solution for unsupervised colorectal polyp segmentation, significantly reducing the dependency on manual annotations while maintaining high segmentation accuracy.

## 1 Introduction

**Figure 1:**
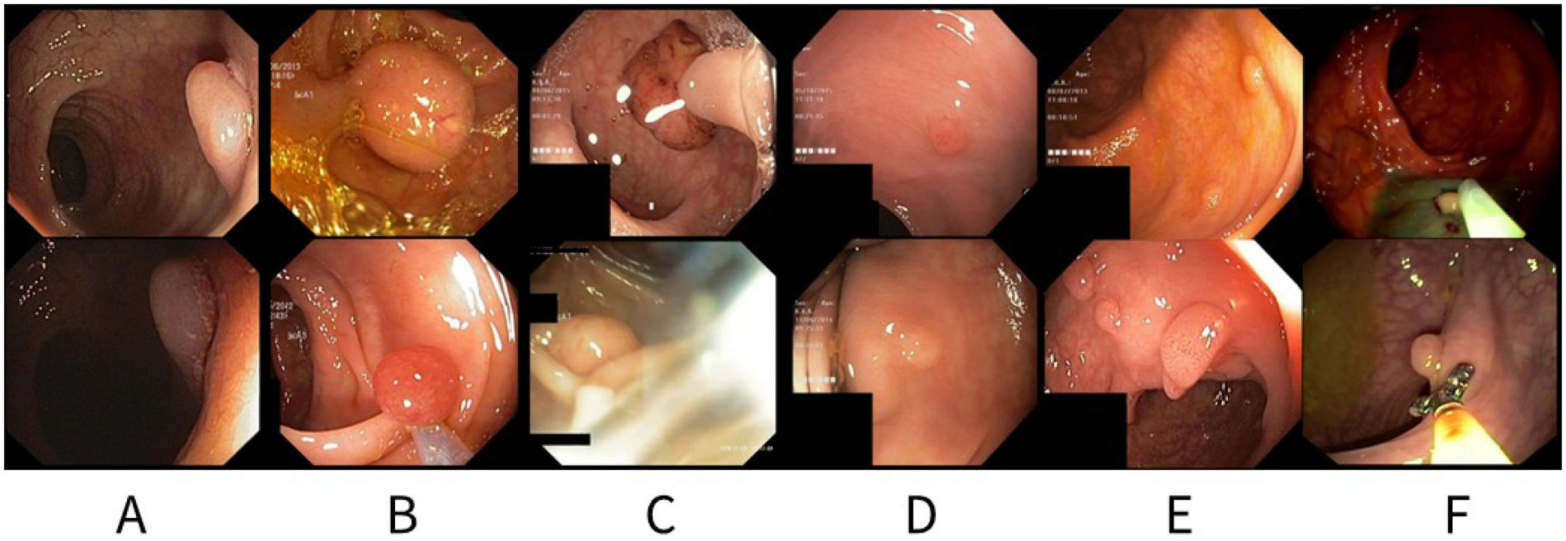
Representative colonoscopy frames illustrating challenges in colorectal polyp segmentation: **(A)** color variations within the same polyp due to lighting changes; **(B)** appearance variability between different polyps; **(C)** visual artifacts such as specular reflections, floating debris, stool, bubbles, and pixel saturation; **(D)** variations in polyp size and shape, with often unclear boundaries between polyp and surrounding mucosa; **(E)** presence of multiple polyps in the same frame; **(F)** occlusion or interference from surgical instruments.

Colorectal cancer (CRC) is the third most common cancer and second leading cause of cancer death worldwide; early detection is crucial, as survival exceeds 90% in early stages but falls to 5% in metastatic stages [1, 2]. Colonoscopy is the gold standard for CRC screening, enabling both visual inspection and removal of precancerous lesions. However, reliable polyp detection remains challenging due to factors (see Figure 1) such as variable illumination and appearance, artifacts, indistinct boundaries, multiple polyps or surgical tools in view, and anatomical variability, which hinder both manual and automated analysis [3–5].

In response to these challenges, systems based on Artificial Intelligence, and in particular Deep Learning (DL), have emerged as promising tools to enhance the precision and consistency of polyp detection and segmentation during colonoscopy, reducing polyp miss rates by up to 50%, compared to conventional visual inspection alone [6, 7]. Although automatic detection and classification are essential to assess polyp presence and risk, precise boundary delineation is equally important, as size and morphology guide malignancy prediction and the choice of resection technique [3, 8].

Traditional supervised DL models have consistently demonstrated high accuracy in colorectal polyp segmentation, however, their reliance on extensive, meticulously annotated datasets poses significant limitations in contexts where expert labeling is scarce, time-consuming, or prohibitively expensive [3, 8–10]. In response, unsupervised methods have emerged as a potential alternative, exhibiting encouraging performance in polyp detection tasks [11–13]; nonetheless, their extension to segmentation remains in its nascent stages [3, 4]. Unsupervised segmentation offers key advantages for clinical use [12]: (1) training data are easy to obtain due to the abundance of normal colonoscopy images; and (2) it avoids the need for annotated datasets covering all abnormal subtypes, including rare or early-stage lesions. Despite these advantages, the high variability of colorectal mucosa remains a major challenge for unsupervised segmentation. As an example, on Hyper-Kvasir [14], supervised methods such as PraNet, Polyp-PVT, and TransNetR achieve Dice and IoU values higher than 0.89 and 0.80, respectively, whereas recent unsupervised approaches, such as PMSACL–PaDiM (0.406 IoU, 0.554 Dice) and MemMC–MAE (0.419 IoU), remain well below supervised performance, highlighting the need for more effective unsupervised paradigms [11, 12, 15–17].

Among unsupervised paradigms, Unsupervised Anomaly Detection (UAD) addresses the challenging task of identifying samples that deviate from normal patterns, assuming abundant normal data and scarce, heterogeneous anomalies that are difficult to collect comprehensively. Consequently, most anomaly detection methods are trained exclusively using normal data [18].

This work proposes and validates an UAD solution called MAPSeg (Memory-Augmented Polyp Segmentation), a framework for unsupervised polyp detection which does not require real polyp samples, segmentation masks, or handcrafted priors. Specifically, following the formulation of Cai et al. [18], MAPSeg is configured as a self-supervised one-stage approach trained to detect synthesized anomalies to be subsequently applied directly to real anomaly detection, thus reformulating the unsupervised problem into a proxy supervised objective through the construction of synthetic anomalies from normal data. Self-supervised learning (SSL) refers to learning paradigms in which supervisory data are derived automatically from the data itself, without requiring manual annotations. In anomaly detection, SSL often relies on proxy tasks or synthetically generated perturbations that enable the model to learn meaningful representations of normality and deviation patterns from unlabeled data. In particular, the main contribution in the MAPSeg framework is the definition of SIMPO (SIMulation of POlyps) a fully unsupervised technique aimed at synthesizing realistic polyp-like anomalies directly from normal colonoscopy images that represents a fundamental component of MAPSeg. Building on this, MAPSeg leverages a learned memory module to encode prototypical patterns of healthy mucosa, allowing anomalous regions to be identified as structured deviations from this internal representation, without requiring manual annotations.

The contributions of this work are summarized as follows:

- An original domain-specific simulation strategy named SIMPO is presented, which generates realistic polyp-like anomalies from normal data, without relying on real polyp samples, segmentation masks, or handcrafted priors.
- The SIMPO block has been integrated and evaluated within MAPSeg, a memory-guided end-to-end anomaly segmentation framework under a one-class training paradigm without relying on manual annotations.
- A comprehensive evaluation of MAPSeg under both in-distribution and out-of-distribution settings is conducted, using datasets from multiple clinical centers to assess robustness under distributional shift.
- A structured review of UAD methods for colorectal polyp segmentation that do not rely on abnormal samples is provided. Unlike previous surveys that primarily focus on fully- or semi-supervised methods, this work categorizes UAD approaches by learning paradigm and analyzes their practical relevance for clinical deployment.
- A comparison with state-of-the-art UAD methods for colorectal polyp segmentation analyzed during the review, followed by ablation studies to confirm the effectiveness of each component of the proposed approach, is provided.

## 2 Related works

Although several surveys review colorectal polyp segmentation, they mainly focus on fully supervised methods [3, 4, 19–21]. Broader reviews on unsupervised medical image segmentation [22–24] provide general taxonomies but do not specifically address UAD approaches for colorectal polyp segmentation, for which no dedicated review currently exists. To address this gap, the present work offers a focused and comparative analysis of UAD-based strategies for unsupervised colorectal polyp segmentation that rely only on real normal samples. A systematic search was conducted in both Scopus and Google Scholar databases, targeting papers that included in their title, abstract, or keywords matching the following query: [(“unsupervised anomaly detection” OR “unsupervised anomaly segmentation”)] AND (“colorectal polyp segmentation”). Only studies consistent with the objectives of the present work were included. Based on these criteria, the following representative UAD methods were selected for analysis and comparison, as summarized in Table 1: PaDiM [25], IGD [26], CCD [13], PMSACL [12], and MemMC-MAE [11]. Considering the performance reported in Table 1, it can be observed how SoA UAD methods based solely on normal samples still achieve significantly lower performance compared to supervised approaches. Hereafter, these approaches are briefly described according to the taxonomy proposed by Cai et al. [18], which categorizes the existing anomaly detection approaches into three primary paradigms: reconstruction-based, SSL-based and feature reference-based.

**Table 1:** Selected UAD methods for colorectal polyp segmentation on the Hyper-Kvasir dataset, categorized according to the taxonomy proposed by Cai et al. The reported IoU and Dice scores correspond to the results presented in the original papers.

Reference	Method	Taxonomy	IoU	DICE	Publicly available code
Defard et al. [25]	PaDiM	Feature reference-based	0.341*	0.475*	Yes
Chen et al. [26]	IGD	Reconstruction-based	0.303*	0.417*	Yes
Tian et al. [13]	CCD+IGD	Self-supervised/Reconstruction-based	0.372	0.502*	Yes
Tian et al. [12]	PMSACL+PaDiM	Self-supervised/Reconstruction-based	0.406	0.554	No
Tian et al. [11]	MemMC-MAE	Reconstruction-based	0.419	–	No

Results marked with an asterisk (*) are directly taken from [12].

### 2.1 Reconstruction-based anomaly detection

These methods typically employ generative models trained to reconstruct only normal samples [27–29]. During inference, the reconstruction error is used as an anomaly score: regions with high reconstruction errors are considered anomalous. This paradigm relies on the assumption that models trained exclusively on normal data will fail to accurately reconstruct unseen abnormalities. MemMC-MAE extends the MAE framework by reconstructing masked image regions and deriving anomaly scores from reconstruction errors [11]. Also IGD belongs to this category, but, as a feature-reconstruction method, it derives anomaly signals from inconsistencies in the latent space rather than pixel-level reconstruction errors [26].

### 2.2 Self-supervised learning-based anomaly detection

SSL has emerged as a powerful strategy for anomaly detection, enabling models to learn useful representations from unlabeled data through pretext tasks. The one-stage approach considers a model that is trained to detect synthetically generated anomalies and then applied to detect real-world anomalies. In contrast, the two-stage approach decouples representation learning from anomaly detection. The first stage involves training a network on a pretext task, such as contrastive learning, to extract semantically rich representations. The second stage uses a one-class classifier trained to discriminate normal and abnormal features. Tian et al. [13] proposed CCD, that considers an encoder pre-trained via contrastive learning with pseudo-anomalies generated through strong augmentations, and then transferred to downstream detectors such as IGD [26]. More recently, Tian et al. [12] introduced PMSACL, which follows the same two-stage paradigm but employs MedMix augmentations to create pseudo-anomalies and enhances contrastive learning. The pre-trained encoder is subsequently integrated into detectors like IGD or PaDiM [25], enabling effective anomaly detection without real anomalies during training.

### 2.3 Feature reference-based anomaly detection

Feature reference-based methods identify anomalies by computing the discrepancy between current feature representations and reference features, without explicitly reconstructing the input image. These methods typically extract local features from a pre-trained encoder and store a representative set of normal patterns in a memory bank. During inference, the similarity or distance between features of input patches and stored prototypes is computed to detect anomalies. PaDiM [25] extracts multi-scale semantic feature vectors from a frozen pre-trained convolutional encoder, and estimates a multivariate Gaussian distribution for each spatial location across normal training samples. During inference, the Mahalanobis distance between each test patch and its corresponding distribution is computed to derive an anomaly map.

Among the three categories presented above, the proposed MAPSeg framework falls within self-supervised learning–based methods and adopts a one-stage self-supervised strategy. Its key contribution is the SIMPO strategy, which is specifically designed to mimic clinically relevant polyp characteristics, taking into account visual patterns commonly assessed in clinical practice, i.e., those described by the Narrow-Band Imaging International Colorectal Endoscopic (NICE) classification. Furthermore, SIMPO is integrated into an encoder–decoder architecture with a memory bank, consistent with similar designs previously adopted in different tasks. However, it is important to emphasize that the SIMPO component is not tied to a specific architecture and can be integrated into other anomaly detection frameworks, potentially enhancing their ability to model structured polyp-like lesions in endoscopic images.

## 3 The MAPSeg framework

### 3.1 Design overview

**Figure 2:**
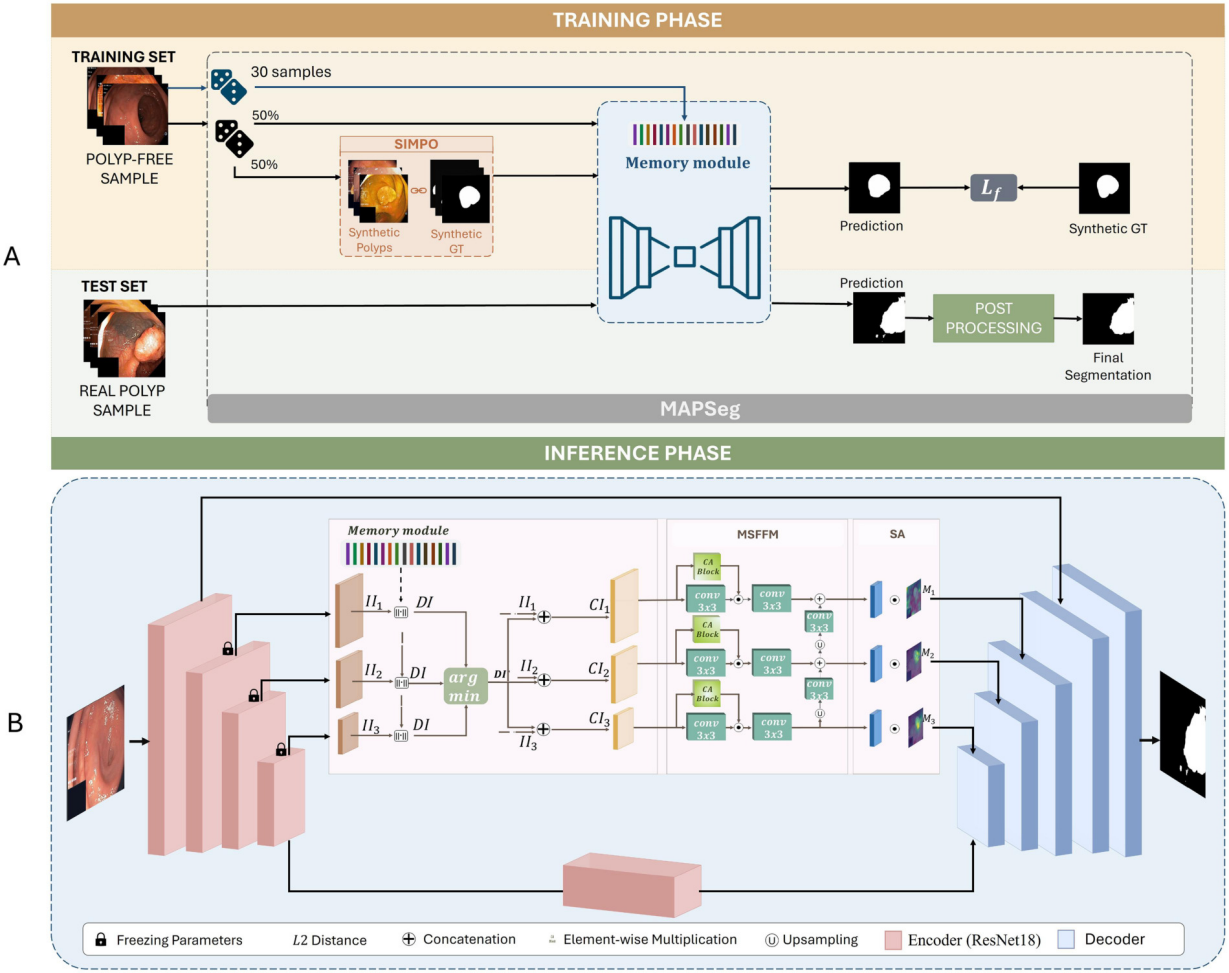
Overview of the proposed MAPSeg framework. **(A)** During the Training Phase, a memory bank is first constructed using polyp-free images to model normal anatomical patterns. The network is then trained using a combination of original normal images and SIMPO-generated synthetic polyp samples. For each input, feature representations are compared with the memory bank to compute discrepancy maps that highlight abnormal regions. The refined features are decoded to produce segmentation masks, optimized using Focal Loss. During the Inference Phase, unseen polyp images are processed using the fixed memory bank and the same discrepancy-guided mechanism, followed by decoding and post-processing to generate the final segmentation masks. **(B)** Detailed architecture of the memory-augmented Convolutional Neural Network (blue module in A), including the memory matching mechanism, discrepancy selection via minimum L2 distance, MSFFM, spatial attention (SA) blocks, and the decoder with progressive upsampling.

The overall MAPSeg framework is represented in Figure 2A. The design of MAPSeg, drawing inspiration from MemSeg [30], is based on a unified objective: modeling structured normality and leveraging controlled synthetic deviations to enable direct pixel-level anomaly segmentation. To this end, MAPSeg combines a memory-augmented encoder, which captures prototypical representations of healthy mucosa, with SIMPO, a domain-specific strategy that generates anatomically plausible synthetic polyps within realistic colonoscopic contexts.

The memory module stores representative embeddings of normal patterns and acts as a structured prior during training and inference. For each input, feature maps are compared with the memory bank to compute discrepancy maps that highlight deviations from the closest normal prototype. These cues are refined through multi-scale feature fusion and spatial attention before being decoded into the final segmentation mask.

SIMPO provides the supervision required for end-to-end learning without real annotations by introducing anatomically coherent synthetic lesions, ensuring that learned discrepancies reflect meaningful pathological variations. Training is guided by the Focal Loss function to address class imbalance, and a lightweight post-processing step enhances morphological consistency of the predictions.

### 3.2 The MAPSeg architecture

MAPSeg is a memory-augmented encoder-decoder framework designed to model normal anatomical priors and localize structured deviations at multiple feature scales. An overview of the architecture is illustrated in Figure 2B.

#### 3.2.1 Encoder and feature extraction

A ResNet18 backbone is employed as the encoder to extract multi-scale feature representations from input images resized to 256 × 256. Intermediate feature maps at different spatial resolutions are retained to preserve both high-level semantic information and fine-grained spatial details.

#### 3.2.2 Memory-guided normality modeling

To explicitly encode healthy anatomical patterns, MAPSeg incorporates a fixed memory module constructed from a small subset of N polyp-free images that are randomly selected from the training dataset. These images are passed through the encoder to generate representative feature embeddings, that serve as prototypes of normal mucosa. This stage is performed once at the beginning and is not updated further.

During both training and inference, the feature maps of a given input image are compared to the stored memory embeddings using an L2 distance. For each scale, a set of difference maps DI is computed according to Equation (1):

$$\mathrm{DI}=\overset{N}{\underset{i=1}{⋃}}\|{\mathrm{MI}}_{i}-\mathrm{II}{\|}_{2}$$ (1)

where $\|{\mathrm{MI}}_{i}-\mathrm{II}{\|}_{2}$ denotes a difference map measuring the pixel-wise deviation between the II, i.e., input sample embedding, and the i-th sample memory embedding MI. To identify the most similar reference pattern in memory, the system selects the map ${\mathrm{DI}}^{*}$ featuring the minimum total sum of pixel-wise differences computed according to Equation (2):

$${\mathrm{DI}}^{*}=\arg\min_{{\mathrm{DI}}_{i}\in \mathrm{DI}}\sum_{x\in {\mathrm{DI}}_{i}}x,\;i\in [1,N]$$ (2)

where x is pixel-level difference.

#### 3.2.3 Attention and multi-scale feature fusion

The selected ${\mathrm{DI}}^{*}$ highlights deviations from normality by measuring the discrepancy between the input image and its closest memory embedding. Higher values in ${\mathrm{DI}}^{*}$ correspond to more anomalous regions. At each encoder scale, ${\mathrm{DI}}^{*}$ is concatenated with the input features II along the channel dimension, producing the combined feature maps ${\mathrm{CI}}_{1}$, ${\mathrm{CI}}_{2}$, and ${\mathrm{CI}}_{3}$.

These are processed through a Multi-Scale Feature Fusion Module (MSFFM). This module performs:

1. A 3×3 convolution to reduce feature redundancy and harmonize the semantic space.
2. Recalibration, within each ${\mathrm{CI}}_{n}$, of spatial and channel-wise relationships is achieved through Coordinate Attention (CA) blocks.
3. Multi-scale alignment via upsampling and convolution, followed by element-wise addition across scales to generate fused features.

To further enhance localization, the spatial attention maps $M_{3}$, $M_{2}$, and $M_{1}$ are derived from the difference maps at each resolution by averaging its channels at each resolution using the Equation (3):

$$M_{3}=\frac{1}{C_{3}}\sum_{i=1}^{C_{3}}{\mathrm{DI}}_{3i}^{*}\;M_{2}=\frac{1}{C_{2}}\sum_{i=1}^{C_{2}}{\mathrm{DI}}_{2i}^{*}⊙M_{3}^{↑}\;M_{1}=\frac{1}{C_{1}}\sum_{i=1}^{C_{1}}{\mathrm{DI}}_{1i}^{*}⊙M_{2}^{↑}$$ (3)

where $M_{n}^{↑}$ denotes the upsampled attention map from the previous scale, and ⊙ indicates element-wise multiplication. Finally, the fused features are modulated by the attention maps $M_{n}$ and passed to the decoder to produce the final segmentation mask.

#### 3.2.4 Decoder and segmentation output

The refined multi-scale representations are forwarded to a decoder with progressive upsampling, which integrates discrepancy-guided attention and multi-scale fusion to translate structured feature deviations into the final pixel-level segmentation mask.

### 3.3 SIMPO: generation of synthetic polyps

SIMPO is a novel strategy designed for generating anatomically plausible synthetic polyps directly from real negative colonoscopy images. SIMPO is specifically tailored to the visual and structural characteristics of the colonic environment and operates without learned parameters or supervised generative models, producing synthetic lesions on-the-fly during MAPSeg training, without requiring access to real polyp images or manual annotations.

**Figure 3:**
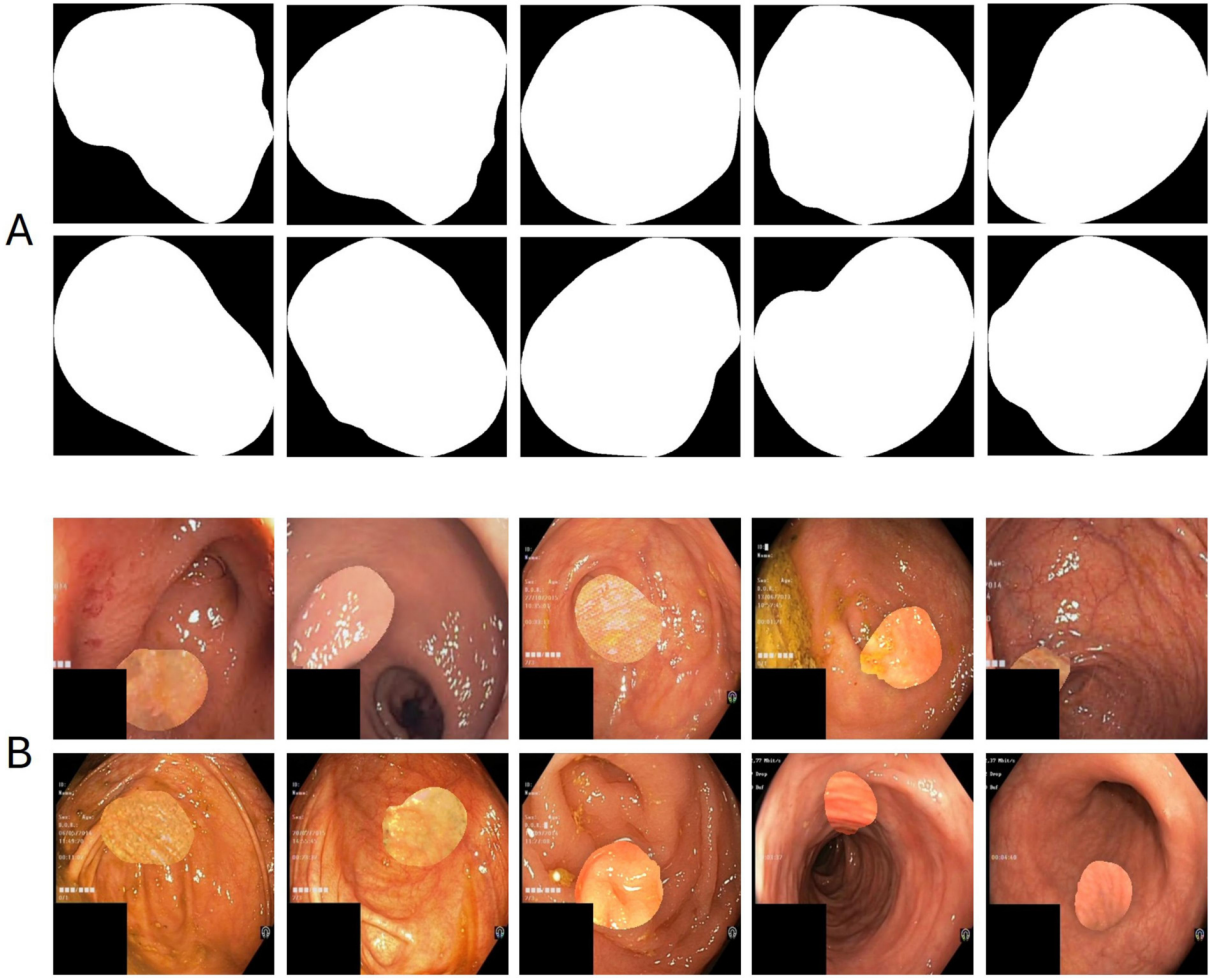
Illustration of examples of masks **(A)** and polyps **(B)** generated.

SIMPO is structured around a sequence of modular processing blocks, each responsible for a specific step in the generation of synthetic masks and polyps (see Figures 3A, B).

**Figure 4:**
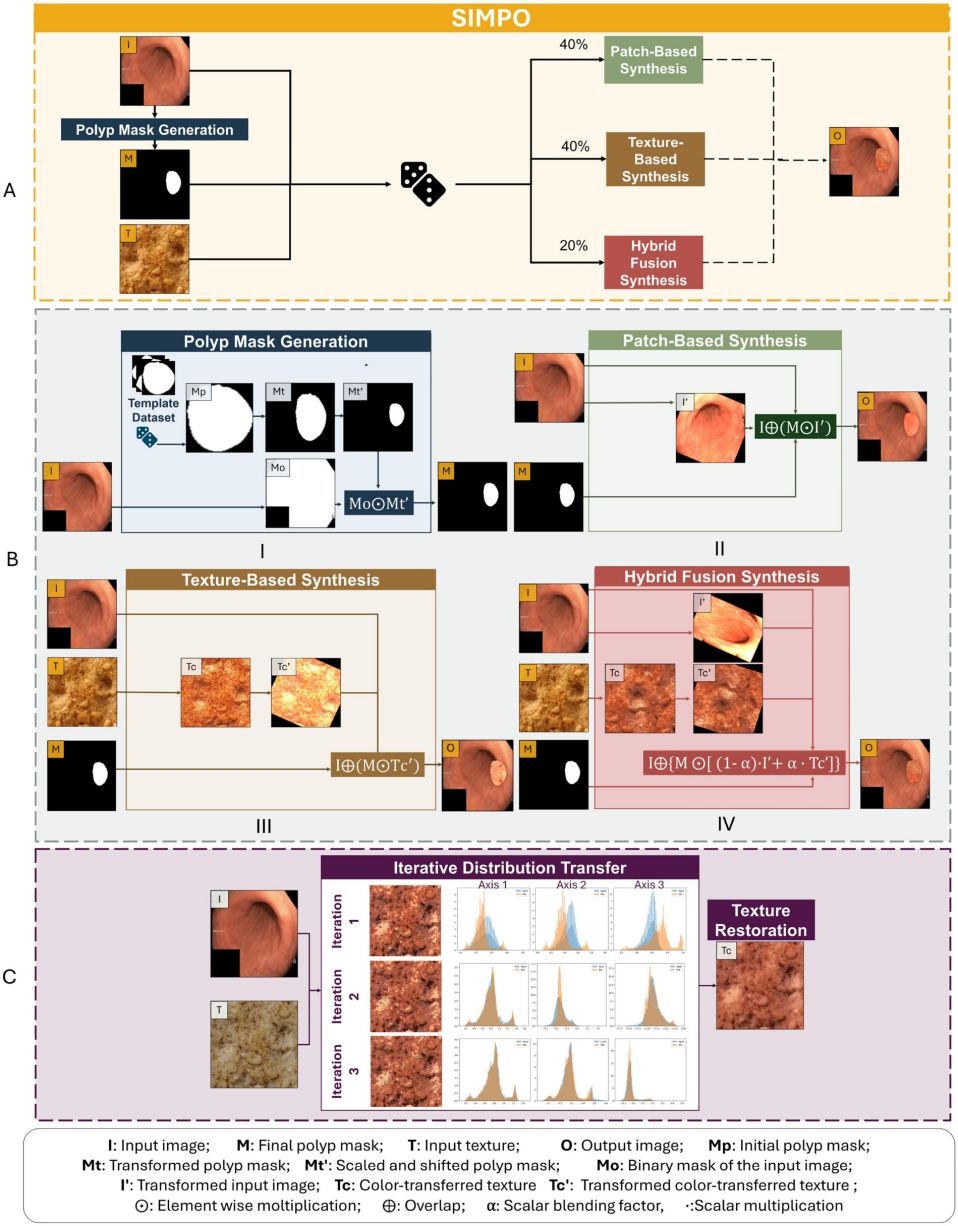
SIMPO pipeline for synthetic polyp generation. **(A)** The architecture selects among three content generation strategies to integrate synthetic polyps into negative colonoscopy images. **(B)** The pipeline components include polyp mask construction from patch-based synthesis using real mucosa patches, texture-based synthesis via adapted external textures, and hybrid fusion combining patch and texture based elements. **(C)** Illustration of the iterative distribution transfer for color adaptation. The image (I) provides color information, while the texture input (T) is the image to be adapted. At each iteration, 1D marginal distributions along three axes are aligned between the input and the reference, progressively reducing distribution mismatch. A final texture restoration step preserves the original structural details while transferring the target color distribution.

The detailed process is illustrated in Figure 4 and begins with the construction of a binary mask that defines the anatomically valid region where the simulated polyp will be inserted, constraining its position, size, and shape. Subsequently, three alternative content generation procedures are defined:

- Patch-based synthesis, which utilizes patches extracted from the same colonoscopic image;
- Texture-based synthesis, relying on external texture sources;
- Hybrid fusion synthesis, which introduces a hybrid strategy that fuses colon-based and texture-based content to enhance both realism and diversity.

These three synthesis strategies are designed to simulate lesion phenotypes inspired by the NICE classification, as described in Table 2. To balance diversity, the three synthesis modes were applied with respective probabilities of 40%, 40%, and 20%. These proportions were determined empirically through an ablation study, which systematically evaluated the impact of different composition ratios on the model’s training stability and segmentation performance.

**Table 2:** Mapping between SIMPO synthesis strategies and clinically-inspired NICE categories.

SIMPO strategy	NICE type	Visual characteristics	Synthesis mechanism
Patch-based	Type 1	Homogeneous surface, pale or similar color to mucosa, low contrast	Local patch insertion with geometric and photometric transformations, shadow-based blending
Hybrid fusion	Type 2	Brownish coloration, mixed texture patterns, moderate structural irregularity	Convex combination of patch- and texture-based synthesis
Texture-based	Type 3	Dark brown/black tones, irregular and amorphous surface, heterogeneous texture	External texture transfer with color matching and mask-based blending

#### 3.3.1 Polyp mask generation

A dedicated dataset of 1,000 polyp template masks is first pre-generated. During training, for each synthetic polyp instance, a mask is randomly sampled from this set and geometrically transformed to generate a specific new final mask.

##### 3.3.1.1 Polyp mask dataset

The generation of 2D polyp contours is performed in polar coordinates because polyps in real colonoscopy videos frequently appear as approximately circular or elliptical structures with irregular boundaries. By perturbing the radius of a base circle through sinusoidal components with varying frequencies and amplitudes, it becomes possible to produce shapes that emulate the soft and complex outlines characteristic of polyps. Let r(θ) denote the radius as a function of the angle θ, as defined in Equation (4):

$$r(\theta )=1+\sum_{i=1}^{n}\xi_{i}\omega_{i}\sin(i\theta +\phi_{i}),\;\theta \in [0,2\pi ]$$ (4)

where:

- n=1000 is the number of harmonics used,
- $\phi_{i}∼U(0,2\pi )$ are random phase shifts,
- $\omega_{i}=1/i^{2}$ are amplitude coefficients decaying quadratically with frequency (Brownian noise),
- $\xi_{i}∼U(0,1)$ is a global scaling factor controlling overall shape deformation.

Brownian noise was selected because of its ability to produce smooth and natural-looking variations, suppressing high-frequency oscillations that would generate unrealistic sharp edges. For each generated mask $M_{p}$, a new set of random $\phi_{i}$ and $\xi_{i}$ is sampled, resulting in unique and highly variable contour shapes. The contour is discretized using 50 2D points obtained by evaluating 50 radius values at equally spaced angles θ over the interval [0,2π]. To obtain smooth and continuous boundaries, cubic spline interpolation is applied, increasing the sampling density by a factor of 10. Finally, the coordinates are converted to Cartesian space and re-scaled to fit the image grid of 512×512 pixels.

##### 3.3.1.2 Polyp mask transformation

As shown in Figure 4B-I, as a first step, a random template mask $M_{p}$ is selected from the mask dataset. To introduce variability and promote generalization, the initial mask is first downscaled by 40%, then subjected to geometric transformations (including rotations, translations, and elastic deformations) resulting in transformed masks $M_{t}$. Subsequently, $M_{t}$ is scaled by a random scaling factor sampled uniformly from the range [0.2,0.9], producing the final mask $M_{t}^{\prime }$. To correctly position the mask within the input image I, binarization via intensity-based thresholding is first applied, obtaining $M_{o}$, which separates mucosal tissue from background and/or possible overlay artifacts. The final valid polyp mask M is computed as the element-wise product $M=M_{0}\cdot M_{t}^{\prime }$. This binary mask ensures that placement occurs strictly within consistent anatomical areas.

#### 3.3.2 Patch-based synthesis

The patch-based synthesis (see Figure 4B-II) strategy is designed to emulate polyp phenotypes typically associated with Type 1 lesions according to the NICE classification, which correspond to hyperplastic or sessile serrated polyps. These lesions are characterized by homogeneous surface patterns and colorations that appear same or slightly lighter than the surrounding mucosal background [31].

The base image I undergoes a sequence of geometric transformations, including horizontal and vertical flipping, random affine scaling along the x-axis, and random rotations. To enhance photometric realism and simulate the appearance of NICE Type 1 polyps, additional colorimetric transformations are applied to obtain $I^{\prime }$: intensity scaling, gamma contrast adjustment, hue and saturation modifications, and Gaussian blurring, with specific transformation ranges summarized in Table 3. Subsequently, the simulated polyp is generated by applying the mask M to the image $I^{\prime }$. To produce a 3D effect and smooth boundary blending between the synthetic lesion and surrounding mucosa, a shadow effect is created by applying a Gaussian blur to the mask, producing a soft transition around its edges. The image is then darkened proportionally to this blurred mask, resulting in a soft shadow focused along the contour. Finally, the synthetic image O is constructed by overlaying the transformed polyp content $I^{\prime }$ into the original image I using the refined mask M.

**Table 3:** Geometric and colorimetric transformations applied during synthetic polyp generation (SIMPO).

Transformation	Operation	Range	Probability
Geometric	Horizontal flip	–	0.5
	Vertical flip	–	0.5
	Rotation	$\left[-90^{\circ },+90^{\circ }\right]$	1.0
	Anisotropic scaling	[0.65,1.0]	0.5
Color/texture	Multiply (Intensity)	(1.5,2.0)	1.0
	Gamma contrast	(0.5,1.5)	1.0
	Gaussian blur	σ=5	0.5
	Add to hue/saturation	[−10,+10]	0.5

#### 3.3.3 Texture-based synthesis

The texture-based synthesis strategy (see Figure 4B-III) aims to reproduce characteristics typical of NICE Type 3 lesions, which are frequently associated with deep submucosal invasive cancers. These lesions often exhibit darker brown to black coloration relative to the background mucosa, patchy whitish areas and amorphous surface patterns [31].

To emulate such complex and irregular appearances, external texture samples from the KTH-TIPS2 dataset [32] are employed as content sources T. Then, the transformed version $T_{c}$ is generated to reproduce the color characteristics of the reference while preserving the original textural structure. Such color transfer is performed using the method proposed by Pitié et al. [33], an iterative approach that progressively aligns color distributions through one-dimensional projections and cumulative distribution matching (see Figure 4C).

The Tc undergoes the geometric transformations reported in Table 3 to obtain $T_{c}^{\prime }$. The intersection between $T^{\prime }$ and the M is first computed, followed by the application of the same soft shadowing effect presented above, enabling smooth boundary blending with the surrounding mucosa. The resulting output is then overlaid onto the original colonoscopy frame I.

#### 3.3.4 Hybrid fusion synthesis

The hybrid fusion synthesis (see Figure 4B-IV) mode aims to simulate the visual characteristics of NICE Type 2 adenomatous polyps, where lesion regions exhibit brownish tones compared to the surrounding mucosa and complex surface patterns.

This method is the fusion of the Path-Based and Texture-Based Synthesis methods. The final synthesized image O is obtained, as defined in Equation (5), by filling the mask M with a convex combination of the transformed images $I^{\prime }$ and $T_{c}^{\prime }$ produced from Path-Based and Texture-Based Synthesis methods, respectively, while the background retains the original image I:

$$O=I⊕M⊙\left[(1-\alpha )\cdot I^{\prime }+\alpha \cdot T_{c}^{\prime }\right]$$ (5)

where α∼U(0.3,0.7) is a scalar interpolation factor controlling the combination of the two synthesized contents, ⊙ denotes element-wise multiplication, and ⊕ indicates the overlap. This fusion strategy enforces spatial continuity, depth perception, and color consistency with the surrounding mucosa, thereby preserving clinical plausibility during synthetic lesion insertion.

### 3.4 Training and inference strategy

#### 3.4.1 Training with synthetic supervision

The model is then trained using a combination of polyp-free images and synthetic abnormal images generated via SIMPO.

The training process is guided by the Focal Loss [34], which is particularly suited to address the class imbalance between background and synthetic polyp regions. Unlike standard loss functions, Focal Loss reduces the contribution of easily classified pixels and emphasizes learning on difficult regions, thereby improving segmentation performance in challenging and highly imbalanced settings. Let $S\in \{0,1{\}}^{H\times W}$ be the binary ground truth mask of the simulated lesion and $\hat{S}\in [0,1{]}^{H\times W}$ the predicted probability map. The loss is computed pixel-wise according to Equation (6):

$$L_{\;f}=-\alpha_{t}(1-p_{t}{)}^{\gamma }\log(p_{t})$$ (6)

where:

- $p_{t}={\hat{S}}_{i,j}$ if $S_{i,j}=1$, and $p_{t}=1-{\hat{S}}_{i,j}$ if $S_{i,j}=0$,
- $\alpha_{t}$ and γ are hyperparameters to control the degree of weighting.

#### 3.4.2 Inference phase

At inference time, only real colonoscopy images are provided. The same encoder–memory comparison mechanism is applied to compute discrepancy maps relative to stored normal prototypes. These discrepancy cues are propagated through the fusion and decoding stages to generate the final segmentation mask.

An optional lightweight post-processing step is applied to improve morphological consistency and suppress spurious detections.

##### 3.4.2.1 Post-processing module

In clinical image segmentation, even minor boundary irregularities or spurious predictions can affect quantitative assessment and diagnostic reliability; therefore, to refine the raw masks, a lightweight post-processing pipeline is proposed and applied to improve morphological consistency and clinical interpretability. Specifically, a morphological closing operation with a 5×5 structuring element removes small holes and smooths object boundaries, followed by hole filling via binary connected component analysis to ensure fully enclosed lesion regions. To suppress residual noise, external contours are extracted and only the largest connected component—assumed to represent the primary polyp—is retained, while smaller components are discarded. This procedure reduces false positives and stabilizes segmentation outputs in complex scenes with minimal computational overhead.

## 4 Experiments

This section describes the experimental protocol adopted to evaluate MAPSeg, including dataset splits, bias mitigation strategies, evaluation protocols, implementation details, and statistical analysis.

### 4.1 Datasets

In this study, multiple publicly available colorectal polyp datasets are employed to evaluate the proposed framework (see Table 4 for a detailed summary of their characteristics). Specifically, the following datasets have been included within the experiments. Hyper-Kvasir [14] is a large-scale gastrointestinal image dataset comprising 110,079 endoscopic images, including 10,662 expert-annotated polyp images with pixel-wise segmentation masks. The dataset contains both normal (polyp-free) and abnormal (polyp-containing) samples. ETIS-LaribPolypDB [35] consists of 196 colonoscopy images containing polyps, each provided with a corresponding pixel-wise annotation. CVC-ClinicDB [36] includes 612 colonoscopy images with pixel-level polyp segmentation masks. CVC-ColonDB [37] comprises 300 colonoscopy images annotated at pixel level for polyp segmentation. The SUN database [38] contains colonoscopy video recordings from which annotated polyp and non-polyp frames were extracted. The refined SUN-SEG dataset [39] reorganizes these recordings into 1,106 short video clips with pixel-wise annotations and provides both polyp and non-polyp frames. The dataset is further divided into Easy and Hard subsets to reflect increasing segmentation difficulty.

**Table 4:** Polyp segmentation datasets used for experiments. Training is performed exclusively on polyp-free images from Hyper-Kvasir. In-distribution evaluation is conducted on the Hyper-Kvasir test split, while all other datasets are used exclusively for out-of-distribution testing.

Dataset	Year	Type	Train size	Test size	Annotation	Data usage
In-distribution						
Hyper-Kvasir	2020	Image-level	1600	1000	Pixel-wise	Train + Test
Out-of-distribution						
Hyper-Kvasir	2020	Image-level	1600	–	Pixel-wise	Train only
ETIS-Larib	2014	Image-level	–	196	Pixel-wise	Test only
CVC-ClinicDB	2015	Image-level	–	612	Pixel-wise	Test only
CVC-ColonDB	2015	Image-level	–	380	Pixel-wise	Test only
SUN-SEG Easy	2022	Video-level	–	544	Pixel-wise, Bounding-box, Scribble, Polygon	Test only
SUN-SEG Hard	2022	Video-level	–	1080	Pixel-wise, Bounding-box, Scribble, Polygon	Test only

In detail, the Hyper-Kvasir dataset was used for both training and in-distribution testing. Out-of-distribution performance was evaluated on external datasets, i.e., CVC-ClinicDB, CVC-ColonDB, SUN-SEG (Easy and Hard), and ETIS-LaribPolypDB. The corresponding dataset splits are summarized in Table 4.

#### 4.1.1 Dataset bias

Hyper-Kvasir contains a known visual bias: normal images display a green thumbnail in the lower-left corner, whereas abnormal images typically contain a black thumbnail or no thumbnail [18]. To mitigate shortcut learning, a black square is uniformly added to the lower-left corner of all images across all datasets prior to training and evaluation. The same pre-processing step was performed on all the other datasets that were used for the out-of-distribution evaluation.

### 4.2 Comparison with the SoA approaches: in-distribution and out-of-distribution evaluation

The proposed framework has been compared with the SoA approaches designed for UAD for colon polyps: PaDiM [25], IGD [26], CCD+IGD [13], PMSACL+PaDiM [12] and MemMC-MAE [11]. The authors of PaDiM, IGD, and CCD+IGD have made their code publicly available, whereas no implementation could be found for PMSACL+PaDiM and MemMC-MAE. For this reason, two complementary procedures were followed to ensure a fair and consistent comparison: when the code was available, all neural networks were retrained and tested, otherwise, the evaluation experiments were specifically designed to ensure a fair comparison. The dataset splits adopted in this study are summarized in Table 4.

#### 4.2.1 In-distribution evaluation

Hyper-Kvasir dataset was employed both for training and testing. MAPSeg and the comparison methods with available code (CCD+IGD, IGD, and PaDiM) were trained and tested following the protocol of prior studies [11–13, 26]: 1,600 normal images for training and 1,000 abnormal images for testing. Each model was evaluated both with and without the proposed post-processing refinement. In addition, the proposed framework has been compared with the MemSeg approach. The evaluation was carried out by comparing the mean and standard deviation of performance metrics across all images in the test set.

When comparing with PMSACL+PaDiM and MemMC-MAE, since their code was not publicly available, the results reported in the original scientific works, based on a sampling-based evaluation strategy, were considered. Specifically, their assessments are conducted using five subsets of 100 polyp images each, randomly sampled from the Hyper-Kvasir test set.

Hence, the same evaluation protocol was performed to allow a fair comparison with MAPSeg. Fifty independent trials were performed, each generating five groups of 100 polyp images randomly selected from the Hyper-Kvasir test set. This repeated sampling strategy allows for a robust and consistent comparison across multiple experimental runs, and was adopted to account for the evident randomness affecting the results reported in the compared works. In this case, the mean performance metrics were computed across the five groups of 100 polyp images and averaged over the 50 trials.

#### 4.2.2 Out-of-distribution evaluation

An out-of-distribution evaluation was performed on multiple external datasets (CVC-ClinicDB, CVC-ColonDB, SUN-SEG Easy/Hard, and ETIS-LaribPolypDB), covering diverse acquisition settings, devices, and lesion characteristics, thereby providing a robust benchmark for assessing generalization under domain shifts [4].

This evaluation encompassed a comparative analysis against PaDiM, IGD and CCD+IGD. The evaluation was carried out by comparing the mean and standard deviation of performance metrics across all images in the test set for each external dataset. PMSACL+PaDiM and MemMC-MAE were not considered since the respective scientific papers do not report such analysis results.

### 4.3 Performance metrics and statistical analysis

The Intersection-over-Union (IoU) and the Dice Similarity Coefficient (Dice) were used in this work as performance metrics. The IoU, also known as the Jaccard index, and the Dice score are defined in Equation (7):

$$\mathrm{IoU}=\frac{\mathrm{TP}}{\mathrm{TP}+\mathrm{FP}+\mathrm{FN}}\;\mathrm{Dice}=\frac{2\mathrm{TP}}{2\mathrm{TP}+\mathrm{FP}+\mathrm{FN}}$$ (7)

where TP, FP, and FN denote the number of true positive, false positive, and false negative pixels, respectively.

Statistical significance was assessed for both in-distribution and out-of-distribution results by comparing per-image IoU and Dice scores between MAPSeg and each baseline using a paired Student’s *t*-test (SciPy).

### 4.4 MAPSeg implementation details

Considering a batch size set to 8, training is conducted for a maximum of 5,000 epochs using the SGD optimizer with momentum 0.9, weight decay 0.0003, and an initial learning rate of 0.01, decayed according to a cosine annealing schedule. Such number of epochs ensures full convergence under the cosine annealing schedule [30]. Model selection is performed by retaining the checkpoint that maximizes the mean segmentation score, computed as the average of IoU and Dice on the validation set (20% of the training set). In practice, optimal performance is typically reached well before the final epoch.

The training objective uses only the Focal Loss, which addresses class imbalance by emphasizing hard-to-classify pixels. The Focal Loss is configured with a weighting factor α=1 and a focusing parameter γ=4. Key hyperparameters were selected via a grid search. The search explored learning rates in 0.001, 0.01, 0.05, weight decay in $1\times 10^{-4}$, $3\times 10^{-4}$, $5\times 10^{-4}$, optimizer choice (SGD, Adam), and focal loss focusing parameter γ∈{1,2,4}. The complete set of hyperparameters is reported in Table 5.

**Table 5:** Training hyperparameters used for MAPSeg.

Hyperparameter	Value
Input image size	256×256
Batch size	8
Epochs	5000
Optimizer	SGD
Momentum	0.9
Weight decay	0.0003
Initial learning rate	0.01
Learning rate schedule	Cosine annealing
Memory samples	30
γ (Focal loss)	4

MAPSeg contains 19.05M parameters (16.27M trainable) and, when evaluated on an NVIDIA GeForce RTX 4080 with 256×256 inputs and batch size 1, achieves an average latency of 13.16 ms per image (75.99 FPS).

## 5 Results and discussions

This section presents a comparative evaluation of MAPSeg against state-of-the-art methods in both in-distribution and out-of-distribution settings, along with qualitative results. In addition, we conduct ablation studies—systematic experiments in which individual components are modified or removed—to assess the contribution of key design choices, including the synthesis strategy, loss formulation, and memory size.

### 5.1 Comparison with existing methods

#### 5.1.1 In-distribution evaluation

Table 6 reports the segmentation performance of all evaluated models without the application of the post-processing step. Among all compared methods, MAPSeg achieves an IoU of 0.48±0.22 and a Dice of 0.61±0.22, outperforming the closest baselines (PaDiM and IGD) by margins of 0.29 in IoU and 0.31 in Dice. Table 7 reports the segmentation performance of all evaluated models with the application of the post-processing step. MAPSeg improves to 0.50±0.25 IoU and 0.63±0.26 Dice. In contrast, competing methods exhibit minimal or inconsistent improvements. In terms of relative improvements, MAPSeg shows superiority over all baselines. All improvements achieved by MAPSeg over competing methods are statistically significant, as confirmed by paired t-tests on per-image IoU and Dice scores (p<0.001).

**Table 6:** Comparison of polyp segmentation performance across different UAD methods without post-processing.

Model	No post-process			
	IoU	Dice	%ΔIoU	%ΔDice
Methods with available code				
MemSeg	$0.09\pm 0.05^{*}$	$0.33\pm 0.16^{*}$	+433%	+85%
PaDiM	$0.19\pm 0.11^{*}$	$0.29\pm 0.15^{*}$	+153%	+110%
IGD	$0.19\pm 0.14^{*}$	$0.30\pm 0.18^{*}$	+153%	+103%
CCD+IGD	$0.17\pm 0.14^{*}$	$0.27\pm 0.18^{*}$	+182%	+126%
**MAPSeg**	0.48±0.22	0.61±0.22	–	–
Methods without available code				
PMSACL-PaDiM	$0.406^{†}$	$0.554^{†}$	+23%	+12%
MemMC-MAE	$0.419^{†}$	–	+19%	–
**MAPSeg**	0.50±0.02	0.62±0.03	–	–

Results are reported as mean ± standard deviation for the IoU and Dice metrics, along with relative improvements (%ΔIoU and %ΔDice) of each baseline method with respect to MAPSeg. Results marked with † are taken directly from the original publications. Statistical significance with respect to MAPSeg is assessed using a paired t-test on per-image scores; * indicates p<0.001.

Bold indicates the best metrics and model.

**Table 7:** Comparison of polyp segmentation performance across different UAD methods with post-processing.

Model	Post-process			
	IoU	Dice	%ΔIoU	%ΔDice
MemSeg	$0.05\pm 0.04^{*}$	$0.21\pm 0.15^{*}$	+900%	+200%
PaDiM	$0.21\pm 0.15^{*}$	$0.20\pm 0.17^{*}$	+138%	+215%
IGD	$0.20\pm 0.15^{*}$	$0.30\pm 0.19^{*}$	+150%	+210%
CCD+IGD	$0.06\pm 0.05^{*}$	$0.09\pm 0.08^{*}$	+733%	+600%
**MAPSeg**	0.50±0.25	0.63±0.26	–	–

Results are reported as mean ± standard deviation for the IoU and Dice metrics, along with relative improvements (%ΔIoU and %ΔDice) of each baseline method with respect to MAPSeg. Statistical significance with respect to MAPSeg is assessed using a paired t-test on per-image scores; * indicates p<0.001.

Bold indicates the best metrics and model.

**Figure 5:**
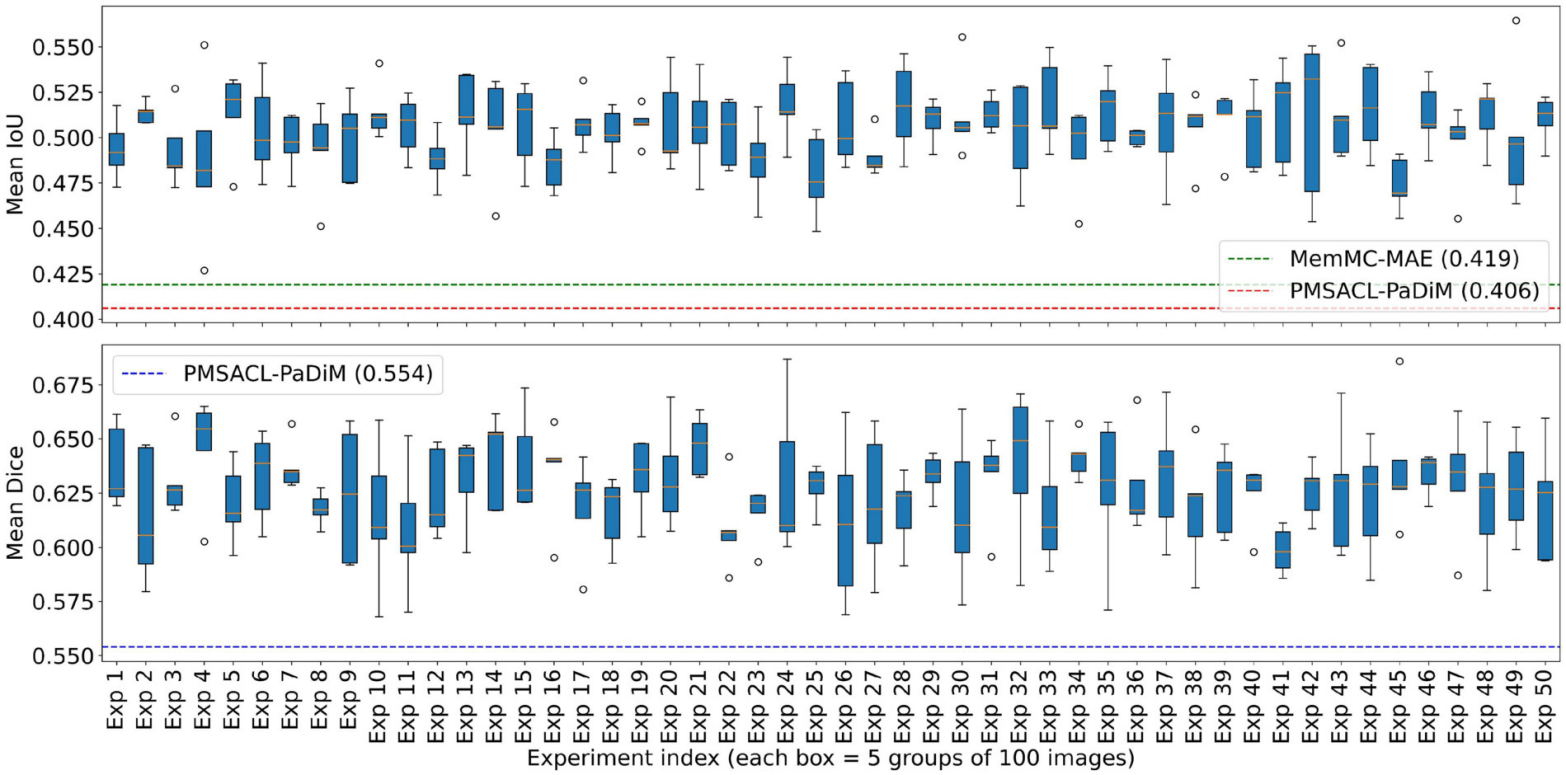
Distribution of MAPSeg performance across 50 trials using the sampling-based evaluation protocol. Each box represents the distribution of mean segmentation scores (computed over 5 groups of 100 images) for a single trial. The top plot shows the distribution of mean IoU scores, while the bottom plot shows the distribution of mean Dice coefficients. Dashed horizontal lines indicate the reference scores reported for PMSACL-PaDiM and MemMC-MAE.

It is worth remembering that to ensure comparability with PMSACL-PaDiM and MemMC-MAE, which report results from a single evaluation over five image groups, the same protocol was reproduced. Hence, MAPSeg was evaluated over 50 independent runs on randomly sampled subsets, reporting mean performance and standard deviations. As illustrated in Figure 5, MAPSeg achieves 0.50±0.02 IoU and 0.62±0.03 Dice, exceeding PMSACL-PaDiM (0.406 IoU, 0.554 Dice) and MemMC-MAE (0.419 IoU) consistently across runs.

#### 5.1.2 Out-of-distribution evaluation

Table 8 reports the segmentation performance of MAPSeg and representative UAD baselines (PaDiM, IGD, and CCD+IGD) across out-of-distribution datasets, measured in terms of mean IoU and Dice with standard deviations. On CVC-ClinicDB, MAPSeg achieves 0.34±0.22 IoU and 0.47±0.24 Dice, while PaDiM and CCD+IGD obtain 0.12±0.09 and 0.04±0.08 IoU, respectively. On CVC-ColonDB, MAPSeg reports a Dice score of 0.34±0.21, whereas CCD+IGD decreases to 0.04±0.11. On the SUN-SEG datasets, MAPSeg achieves 0.38±0.27 and 0.38±0.26 IoU on the Easy and Hard subsets, respectively, with corresponding Dice values of 0.49±0.29. In contrast, IGD and CCD+IGD show notable performance degradation, particularly on the Hard subset. Finally, on the ETIS-Larib dataset, MAPSeg achieves 0.21±0.22 IoU and 0.30±0.27 Dice, while competing methods remain below 0.14 in both metrics. All improvements of MAPSeg over baseline methods are statistically significant according to paired t-tests on per-image IoU and Dice scores (p<0.001; see Table 8).

**Table 8:** Quantitative segmentation performance on in-distribution (Hyper-Kvasir) and out-of-distribution datasets (CVC-ClinicDB, CVC-ColonDB, SUN-SEG Easy/Hard, ETIS-Larib).

	Hyper-Kvasir		CVC-ClinicDB		CVC-ColonDB	
Model	IoU	Dice	IoU	Dice	IoU	Dice
PaDiM	$0.19\pm 0.11^{*}$	$0.29\pm 0.15^{*}$	$0.12\pm 0.09^{*}$	$0.20\pm 0.14^{*}$	$0.12\pm 0.09^{*}$	$0.21\pm 0.13^{*}$
IGD	$0.19\pm 0.14^{*}$	$0.30\pm 0.18^{*}$	$0.14\pm 0.12^{*}$	$0.23\pm 0.15^{*}$	$0.10\pm 0.12^{*}$	$0.17\pm 0.17^{*}$
CCD+IGD	$0.17\pm 0.14^{*}$	$0.27\pm 0.18^{*}$	$0.04\pm 0.08^{*}$	$0.08\pm 0.12^{*}$	$0.03\pm 0.08^{*}$	$0.04\pm 0.11^{*}$
**MAPSeg**	0.48±0.22	0.61±0.22	0.34±0.22	0.47±0.24	0.22±0.20	0.34±0.21

	SUN-SEG (Easy)		SUN-SEG (Hard)		ETIS-Larib	
PaDiM	$0.15\pm 0.11^{*}$	$0.25\pm 0.16^{*}$	$0.16\pm 0.13^{*}$	$0.25\pm 0.18^{*}$	$0.08\pm 0.07^{*}$	$0.14\pm 0.11^{*}$
IGD	$0.12\pm 0.12^{*}$	$0.20\pm 0.16^{*}$	$0.11\pm 0.11^{*}$	$0.18\pm 0.26^{*}$	$0.06\pm 0.07^{*}$	$0.11\pm 0.12^{*}$
CCD+IGD	$0.03\pm 0.06^{*}$	$0.05\pm 0.10^{*}$	$0.03\pm 0.05^{*}$	$0.05\pm 0.08^{*}$	$0.03\pm 0.06^{*}$	$0.05\pm 0.09^{*}$
**MAPSeg**	0.38±0.27	0.49±0.29	0.38±0.26	0.49±0.29	0.21±0.22	0.30±0.27

Results are reported as mean ± standard deviation of IoU and Dice coefficients. Statistical significance with respect to MAPSeg is assessed using a paired t-test on per-image scores; * indicates p<0.001.

Bold indicates the best metrics and model.

### 5.2 Qualitative results

**Figure 6:**
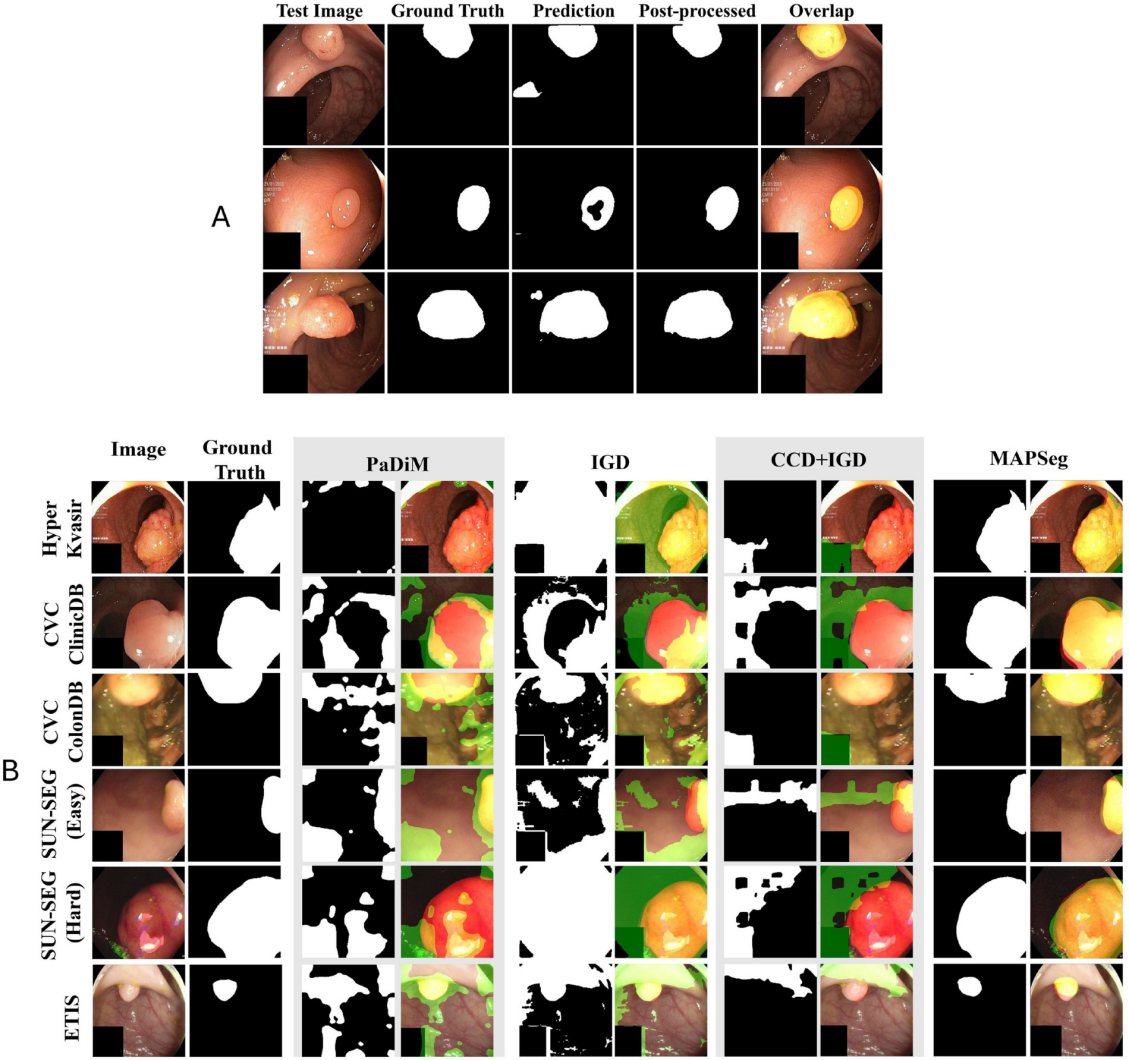
Comparison of colorectal polyp segmentation results. **(A)** Qualitative comparison of MAPSeg segmentation outputs on Hyper-Kvasir samples before and after post-processing. Each triplet shows, from left to right, the original colonoscopy image, the raw MAPSeg prediction, and the refined post-processed overlay. **(B)** Qualitative comparison across different datasets and methods. The leftmost column shows input colonoscopy images from various datasets (top to bottom: Hyper-Kvasir, CVC-ClinicDB, CVC-ColonDB, SUN-SEG (Easy), SUN-SEG (Hard), and ETIS). The second column displays ground truth segmentation masks. The remaining columns report predictions from MAPSeg and three baseline methods (CCD+IGD, IGD, and PaDiM). For each method, predicted regions are shown in green, ground truth masks in red, and overlapping areas in yellow.

Figure 6A illustrates the qualitative effect of post-processing on MAPSeg outputs using representative samples from the Hyper-Kvasir dataset. Raw predictions may exhibit coarse boundaries or minor discontinuities, whereas post-processing produces more compact and smoother masks, consistent with the quantitative improvements reported in Table 7. Figure 6B presents a visual comparison between MAPSeg and representative baseline models on challenging colonoscopy frames. The examples highlight differences in boundary delineation under conditions such as low contrast, specular reflections, and complex mucosal textures.

**Figure 7:**
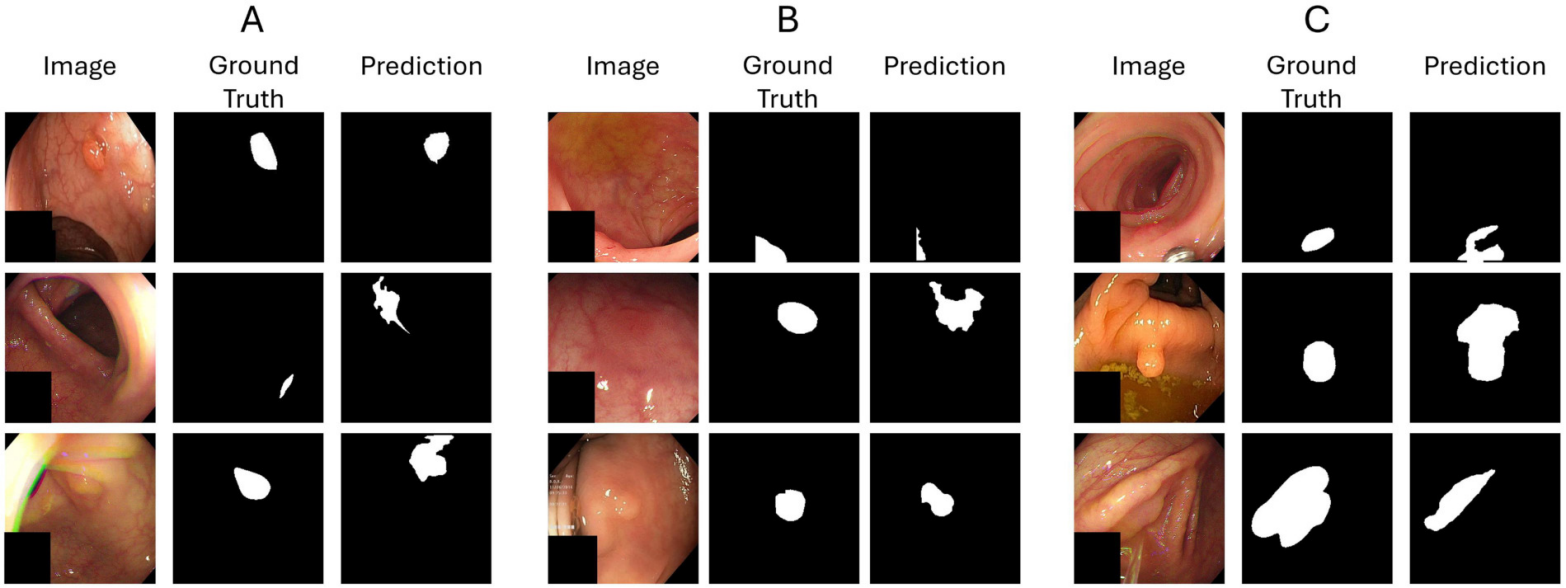
Representative failure modes and suboptimal predictions of MAPSeg under different challenging conditions. **(A)** Small polyps under different illumination conditions. **(B)** Flat or low-contrast lesions, characterized by minimal deviation from surrounding mucosa. **(C)** Visually complex regions affected by artifacts such as specular reflections, mucosal folds, or residual debris.

In addition to these examples, we further analyze typical failure modes and suboptimal predictions of the proposed framework. As illustrated in Figure 7A, challenging scenarios include extremely small polyps, flat or low-contrast lesions (Figure 7B), and visually complex regions affected by artifacts such as specular reflections or debris (Figure 7C).

Notably, Figure 7A highlights that small polyps are particularly prone to missed or incomplete segmentation, likely due to their limited spatial extent and weak visual saliency under varying illumination conditions.

A particularly challenging case is represented by flat or depressed polyps (Figure 7B), which exhibit minimal textural and structural deviation from the surrounding mucosa. Due to this limited contrast, such lesions may be more difficult to distinguish within an anomaly-based framework. Nevertheless, qualitative results suggest that the model is still able to highlight the presence of these lesions, although the resulting predictions may be incomplete or not accurately delineated.

Another aspect to consider is the presence of non-pathological artifacts in endoscopic images, such as specular reflections, bubbles, or fecal debris. These elements are also present in the polyp-free training data (e.g., Hyper-Kvasir) and are therefore incorporated into the memory representation of normal mucosa. However, in visually complex scenarios, such artifacts may variably affect the model predictions: in some cases, they lead to false positive activations in regions that do not correspond to actual lesions, while in others the model correctly ignores them, demonstrating a certain degree of robustness (Figure 7C).

### 5.3 Ablation studies

A series of ablation experiments was conducted to assess the contribution of the framework’s key components, through a systematic analysis in which individual modules are removed or modified to evaluate their impact on overall performance. Specifically, the analysis examines: (i) the impact of different polyp simulation strategies (patch-based, texture-based, and hybrid fusion); (ii) the influence of the loss function design; (iii) the effect of varying the memory sample size on segmentation performance; and (iv) the sensitivity to training set size.

#### 5.3.1 Impact of patch-based, texture-based and hybrid fusion synthesis ratios

The impact of different synthesis modalities was evaluated by varying the proportions of Patch-Based, Texture-Based, and Hybrid Fusion strategies. As shown in Table 9, the best performance is achieved by balancing structural consistency (Patch-Based) and photometric variability (Texture-Based and Hybrid Fusion). Among the different tested combinations, the configuration with 40% Patch-Based, 40% Texture-Based, and 20% Hybrid Fusion achieved the best performance metrics, i.e., 0.48 IoU and 0.61 Dice. Overall, these findings indicate that combining structural and photometric variability is necessary to avoid underfitting (single modality) and instability (excessive blending).

**Table 9:** Ablation studies results. Effect of different synthesis percentages for Patch-Based, Texture-Based, and Hybrid Fusion modalities in the SIMPO framework.

Patch based	Texture based	Hybrid fusion	IoU	Dice
100	0	0	0.26	0.56
0	100	0	$7.99\times 10^{-5}$	$1.50\times 10^{-4}$
0	0	100	0.10	0.16
50	0	50	0.20	0.28
0	50	50	0.25	0.36
50	50	0	0.37	0.49
45	45	10	0.47	0.60
**40**	**40**	**20**	**0.48**	**0.61**
35	35	30	0.34	0.46
30	30	40	0.29	0.40
20	20	60	0.34	0.45

$\lambda_{1}$	$\lambda_{f}$	Loss combination	IoU	Dice
1.0	0.0	L1 only	0.05	0.07
0.8	0.2	L1 + Focal	0.19	0.30
0.6	0.4	L1 + Focal	0.24	0.37
0.4	0.6	L1 + Focal	0.24	0.36
0.2	0.8	L1 + Focal	0.31	0.45
**0.0**	**1.0**	**Focal only**	**0.48**	**0.61**

Memory size			IoU	Dice
15			0.40	0.50
**30**			**0.48**	**0.61**
70			0.37	0.52
140			0.37	0.51

The table reports the corresponding segmentation performance on the Hyper-Kvasir dataset for each composition, showing that the best results are achieved with 40% Patch-Based, 40% Texture-Based, and 20% Hybrid Fusion synthesis. It further compares different combinations of $\lambda_{1}$ (L1 loss weight) and $\lambda_{f}$ (Focal loss weight), and analyzes the impact of memory sample size on segmentation performance. IoU and Dice scores are reported for all experimental settings.

Bold indicates the selected parameter/set of parameters according to the best metrics.

#### 5.3.2 Impact of different losses

The contribution of both $L_{1}$ loss and Focal Loss was evaluated (see Table 9). Experiments indicated that the inclusion of the $L_{1}$ component did not substantially improve localization performance and resulted in slower convergence. In contrast, Focal Loss provided more stable training behavior and higher segmentation accuracy. Based on these observations, the final configuration employs Focal Loss only.

#### 5.3.3 Effect of memory sample size

The number of memory items entails a trade-off between representational capacity and overfitting. An ablation study evaluated performance using 15, 30, 70, and 140 memory samples during inference to identify the optimal size. As reported in Table 9, performance improves when increasing memory samples from 15 to 30, reaching 0.48 IoU and 0.61 Dice, indicating better modeling of normal patterns and anomaly discrimination. However, further increasing memory size to 70 and 140 degrades performance, likely due to redundant or less relevant patterns introducing noise and impairing matching during inference.

#### 5.3.4 Sensitivity analysis to training set size

To assess robustness to training data size, a sensitivity analysis was performed on Hyper-Kvasir using reduced training splits. Two settings were considered: Train-3 (three splits, training on two each time; 1,066 samples per run, three runs) and Train-5 (five splits, training on four each time; 1,280 samples per run, five runs). The test set remained fixed, and models were retrained from scratch for each split. Results are reported in Table 10 as mean and standard deviation across runs.

**Table 10:** Sensitivity analysis to training set size. Segmentation performance of MAPSeg obtained by training the model using different subsets of the training data, while keeping the test set fixed.

Setting	IoU	Dice
Train-3	0.395±0.040	0.508±0.053
Train-5	0.409±0.032	0.509±0.047

Results are reported as mean IoU and Dice coefficients across different random splits.

Reducing the training data slightly degrades performance compared to using the full set. Train-3 achieves 0.395±0.040 IoU and 0.508±0.053 Dice (−8.5%, −10.2%), while Train-5 reaches 0.409±0.032 IoU and 0.509±0.047 Dice (−7.1%, −10.1%). Despite this reduction, the low standard deviations across splits indicate stable behavior, suggesting that the training process is robust to variations in the composition of the training sets.

### 5.4 Additional discussions and limitations

The experimental results indicate that MAPSeg effectively models structured deviations between normal mucosa and anomalous regions under a one-class training paradigm. The consistent performance across in-distribution and out-of-distribution datasets, together with statistically significant margins over representative UAD baselines, suggests that integrating synthetic anomaly simulation with memory-guided feature discrepancy modeling provides a more direct optimization of segmentation compared to reconstruction- or distance-based scoring approaches. The ablation studies further clarify the mechanisms underlying these results.

The proposed framework employs a memory module that enables the network to simultaneously learn the characteristics of normal tissue and relative deviations of potential polyps. However, a slight performance degradation observed when using single-modality synthesis highlights that structural realism alone or texture variability alone is insufficient. Instead, a balanced combination appears necessary to prevent shortcut learning and encourage the model to focus on meaningful deviations from normal anatomy. A potential bias introduced by synthetic polyps may concern variability in shape, color, and texture, as SIMPO cannot fully capture the entire spectrum of real polyp appearances. This limitation is partly probabilistic in nature, since the parameters governing polyp generation depend on random variables, and partly due to the finite number of training epochs, during which only a limited—though diverse—set of synthetic variations can be sampled rather than an exhaustive representation of all possible morphologies.

While these limitations exist, the proposed self-supervised approach offers practical advantages in scenarios where large-scale manual annotation is not feasible due to the time-consuming and expert-dependent nature of pixel-wise labeling. In particular, it can facilitate deployment in resource-limited clinical settings, where expert time for annotation is scarce, and in screening applications for rare pathologies, where annotated data are inherently limited. By reducing the reliance on manual annotations, the method lowers the barrier to adoption and enables broader applicability across diverse clinical environments.

Despite these strengths, some limitations should be acknowledged. The framework relies on synthetic anomalies generated by SIMPO. Although designed to promote anatomical plausibility, synthetic modeling inevitably introduces distributional assumptions. The parametric contour generation and blending strategies may not fully represent the morphological diversity of real colorectal lesions, particularly flat, depressed, or highly irregular phenotypes. However, in this context, synthetic anomalies serve as a proxy to model “non-normal” structures, enabling the system to capture distributional discrepancies rather than precise lesion phenotypes. Nonetheless, performance may decrease when real anomalies differ substantially from the variability encompassed by the simulated patterns.

The current formulation operates on individual frames and does not explicitly exploit temporal information available in colonoscopy videos. Incorporating temporal consistency mechanisms may improve prediction stability and reduce variability. Finally, validation has been conducted on publicly available datasets. Prospective real-time clinical studies are required to determine deployment feasibility and integration within endoscopic workflows.

Building upon the observed failure modes and suboptimal predictions of MAPSeg (Figure 7), several directions for future improvements can be identified. In particular, the reduced sensitivity to extremely small polyps indicates that improved modeling of fine-grained visual details may be beneficial. Similarly, the challenges associated with flat or low-contrast lesions suggest that enhancing the distinction between subtle anomalies and normal mucosa could improve delineation accuracy. This limitation is clinically significant, as flat and depressed polyps are considered challenging lesions to identify even during routine colonoscopy, often requiring careful inspection due to their subtle visual characteristics [40]. Furthermore, the variable behavior observed in the presence of non-pathological artifacts highlights the importance of increasing robustness to visually complex patterns. Addressing these aspects represents a promising direction to further improve the reliability and clinical applicability of the proposed framework.

## 6 Conclusion

This work introduced MAPSeg (Memory-Augmented Polyp Segmentation), a self-supervised, one-stage anomaly detection framework for polyp segmentation in colonoscopy images without the need for real abnormal images. The method combines a memory-augmented encoder with SIMPO (SIMulation of POlyps), a novel augmentation strategy that generates anatomically plausible synthetic polyps from normal colonoscopy images without requiring manual annotations. Unlike existing approaches that rely on cut-and-paste heuristics or reconstruction-based pipelines, MAPSeg performs direct end-to-end segmentation guided by memory-based anomaly localization. Experimental evaluations demonstrated the effectiveness of MAPSeg under both in-distribution and out-of-distribution conditions. On the Hyper-Kvasir benchmark, MAPSeg significantly outperformed unsupervised anomaly detection methods, achieving a mean Intersection over Union of 0.50 and a mean Dice coefficient of 0.63 under sampling-based evaluation. Furthermore, MAPSeg exhibited superior generalization capabilities across multiple external datasets, including SUN-SEG, CVC-ClinicDB, CVC-ColonDB, and ETIS-LaribPolypDB. Ablation studies confirmed the importance of key components such as the hybrid fusion synthesis in SIMPO, the focal loss, and the optimal memory size, all of which contributed to the observed performance gains. Overall, MAPSeg demonstrates that memory-guided anomaly segmentation combined with procedural simulation constitutes a viable fully annotation-free alternative to supervised polyp segmentation frameworks.

## Data Availability

Publicly available datasets were analyzed in this study. This data can be found here: -*hyper-kvasir*: https://datasets.simula.no/hyper-kvasir/ -*CVC-ClinicDB* https://www.kaggle.com/datasets/balraj98/cvcclinicdb -*CVC-ColonDB* https://www.kaggle.com/datasets/longvil/cvc-colondb -*ETIS-LaribPolypDB* https://www.kaggle.com/datasets/nguyenvoquocduong/etis-laribpolypdb -*SUN-SEG* https://github.com/GewelsJI/VPS/blob/main/docs/DATA_PREPARATION.md.
